# Risk of Persistence and Progression of Use of 5 Cannabis Products After Experimentation Among Adolescents

**DOI:** 10.1001/jamanetworkopen.2019.19792

**Published:** 2020-01-24

**Authors:** Jessica L. Barrington-Trimis, Junhan Cho, Esthelle Ewusi-Boisvert, Deborah Hasin, Jennifer B. Unger, Richard A. Miech, Adam M. Leventhal

**Affiliations:** 1 Department of Preventive Medicine, University of Southern California, Los Angeles; 2 Department of Psychology, University of Southern California, Los Angeles; 3 Department of Epidemiology, Columbia University, New York, New York; 4 Institute for Social Research, University of Michigan, Ann Arbor

## Abstract

**Importance:**

While a diverse array of cannabis products that may appeal to youth is currently available, it is unknown whether the risk of persistent cannabis use and progression to higher frequency of use after experimentation differs among cannabis products.

**Objective:**

To estimate the comparative relative risk of experimental use of 5 cannabis products on use status and frequency of use among adolescents during 12 months of follow-up.

**Design, Setting, and Participants:**

In this cohort study, data were collected from 3065 adolescents at 10 high schools in southern California, with baseline data collected in spring 2016, when students were in 11th grade, and 6-month and 12-month follow-up surveys collected in fall 2016 and spring 2017, when students were in 12th grade. Analyses, conducted from April to June 2019, were restricted to 2685 participants who were light or nonusers of any cannabis product (ie, ≤2 days in the past 30 days) at baseline.

**Exposures:**

Number of days of use of each cannabis product (ie, combustible, blunts, vaporized, edible, or concentrated) in the past 30 days at baseline (ie, 1-2 vs 0 days).

**Main Outcomes and Measures:**

Past 6-month use (ie, yes vs no) and number of days of use in the past 30 days at 6-month and 12-month follow-ups for each product.

**Results:**

Of 2685 individuals in the analytic sample, 1477 (55.0%) were young women, the mean (SD) age was 17.1 (0.4) years, and a plurality (1231 [46.6%]) were Hispanic individuals. Among them, 158 (5.9%) reported combustible cannabis use on 1 to 2 days of the past 30 days at baseline, 90 (3.4%) reported blunt use, 78 (2.9%) reported edible cannabis use, 17 (0.6%) reported vaping cannabis, and 15 (0.6%) reported using cannabis concentrates. In regression models adjusting for demographic characteristics and poly–cannabis product use, statistically stronger associations of baseline use with subsequent past 6-month use at the 6-month and 12-month follow-ups were observed for combustible cannabis use (odds ratio, 6.01; 95% CI, 3.66-9.85) and cannabis concentrate use (odds ratio, 5.87; 95% CI, 1.18-23.80) compared with use of blunts (OR, 2.77; 95% CI, 1.45-5.29) or edible cannabis (OR, 3.32; 95% CI, 1.86-5.95) (*P *for comparison < .05); vaporized cannabis use (OR, 5.34; 95% CI, 1.51-11.20) was not significantly different from the other products. In similarly adjusted models, we found the association of cannabis use at baseline with mean days of use at the 6-month and 12-month follow-ups was significantly stronger for cannabis concentrate than for other cannabis products; participants who had used cannabis concentrate on 1 to 2 of the past 30 days at baseline (vs 0 days) used cannabis concentrate a mean of 9.42 (95% CI, 2.02-35.50) more days in the past 30 days at the 6-month and 12-month follow-ups (*P* for comparison < .05).

**Conclusions and Relevance:**

Cannabis control efforts should consider targeting specific cannabis products, including combustible cannabis and cannabis concentrate, for maximum public health consequences.

## Key Points

**Question:**

After experimentation in adolescence, do risks of progression and persistence of cannabis use during a 12-month follow-up period differ among 5 cannabis products?

**Findings:**

In this cohort study of 2685 adolescents with no history of heavy cannabis use, after accounting for polyuse of multiple products, the association of baseline experimental use with persistent use and progression of use of that product during a 12-month follow-up period was significantly stronger for cannabis concentrate than for other cannabis products.

**Meaning:**

The rate of persistence and progression after experimentation among adolescents may be amplified with the use of cannabis concentrate compared with other cannabis products.

## Teaser

This cohort study compares the relative risk of the status and frequency of use of 5 cannabis products following experimental use among adolescents during 12 months of follow-up.

## Introduction

The legalization and commercialization of cannabis have increased the diversity of the types of cannabis products in the marketplace. While combustible cannabis (eg, smoking cannabis in a joint or bong) and edible cannabis (eg, consumption of cannabis-infused food items) date back centuries or more,^1^ other cannabis formulations have more recently become available. Cannabis concentrates (eg, dabbing or use of highly concentrated cannabis products, commonly referred to as wax, shatter, budder, or butane hash oil) first gained popularity around 2010 and were notable for high concentrations of tetrahydrocannabinol (THC), the primary psychoactive ingredient in cannabis.^2^ Levels of THC in cannabis concentrate were 2 to 4 times greater than those found in traditional cannabis products, reaching concentrations greater than 80% THC.^2^ With the recent advent and rapid rise to popularity of personal electronic vaping devices,^3^ they have become another popular vehicle for use of high concentrations of cannabis in the form of dry herb, THC e-liquid solutions, or cannabis concentrate solutions.^4^

Many youth experiment with cannabis during adolescence; in 2018, 43.6% of students in 12th grade reported ever having used cannabis, 35.9% reported using cannabis in the past year, and 22.2% reported using cannabis in the past 30 days.^3^ While most youth subsequently discontinue use, a small but appreciable subset continue using cannabis products and progress to higher levels of use; in 2018, 5.9% of students in 12th grade reported daily use of cannabis products.^3^ The period following experimentation represents a critical juncture during which youth decide to continue or discontinue use of cannabis products.^5,6^ The type of cannabis product used may influence subsequent cannabis use patterns following experimentation owing to differential drug delivery mechanisms and variation in the sensory effects associated with the use of different products. Each cannabis product is available in different formulations with varying levels of THC, elicits different sensory effects because of the method of administration (ie, smoking vs eating vs vaping) and the additives present in the product (ie, flavors in edible and vaping products),^7,8,9^ has different pharmacokinetics of drug absorption,^10^ and varies in adolescents’ ability to access that product and use it without consequence (eg, some methods are easier to conceal).^4,11,12,13^ If certain cannabis products are more reinforcing (ie, leading to dependent use patterns), result in more positive sensory effects, or are more accessible to obtain and use, those cannabis products may pose a greater risk of continued use and warrant a public health response targeting those specific cannabis products. However, whether the potential for abuse differs among products is unknown.

In the current study, we examined the association of cannabis use at baseline with persistent (ie, continued) cannabis use and progression to more frequent cannabis use (ie, increase in the number of days of use) over 1 year of follow-up for 5 different cannabis products (ie, combustible cannabis, blunts, vaporized cannabis, cannabis edibles, and cannabis concentrate) in a prospective cohort of adolescents in southern California. Our primary aim was to determine whether the strength of these associations (ie, the risk of persistent use or progression of use) differed by the type of cannabis product used.

## Methods

### Participants and Procedures

Data were drawn from a prospective cohort survey of students from 10 urban and suburban public high schools in Los Angeles County, California,^14^ beginning in October 2013, when participants were in 9th grade. Variables for this study were initially assessed between January and September 2016, when participants were in 11th grade. This assessment serves as the baseline survey for the current study. The sample included students who completed key measures of cannabis product use and were either not current cannabis users (ie, no use of any cannabis product in the last 30 days at baseline) or light users (ie, use on only 1-2 of the last 30 days at baseline) (Figure). Participants were included if they also provided valid data at the 6-month follow-up (September 2016 to March 2017, when participants were in 12th grade) or 12-month follow-up (January to August 2017, also when participants were in 12th grade). Analyses were conducted from April to June 2019. Medicinal cannabis has been legal in California since 1996. In November 2016, California voted to legalize adult-use (ie, recreational) cannabis, and implementation and licensing of retailers became effective in January 2018.^15^ The institutional review board of the University of Southern California approved this study. Written parental informed consent (or verbal consent when written consent was not obtained) and student assent were obtained for all participants before data collection. This study followed the Strengthening the Reporting of Observational Studies in Epidemiology (STROBE) reporting guideline for cohort studies and the American Association for Public Opinion Research (AAPOR) reporting guideline for surveys.

**Figure.**
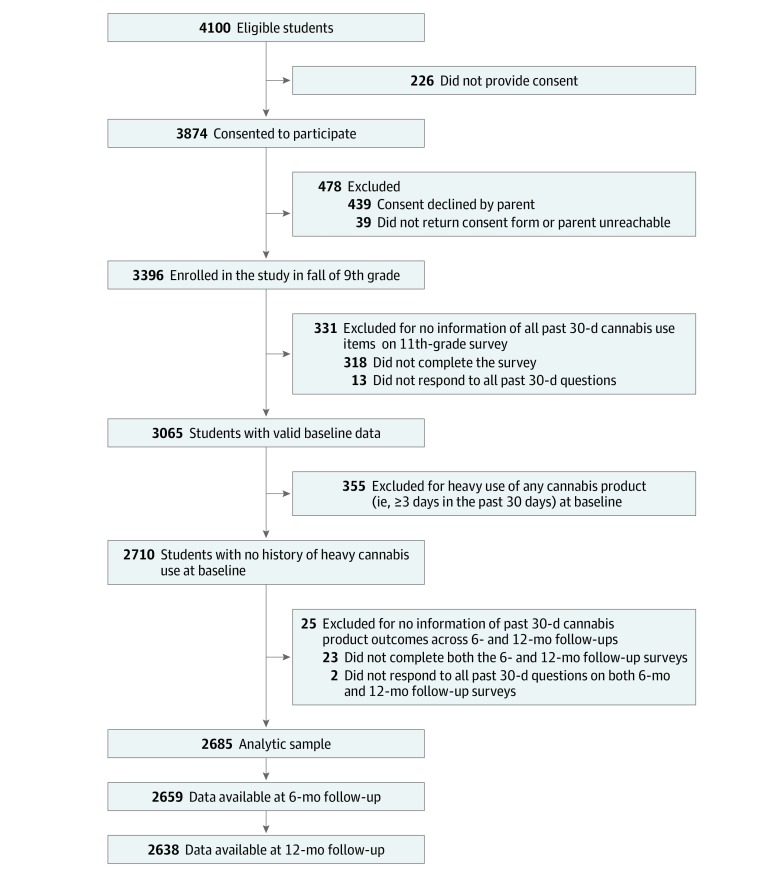
Study Accrual Flow Chart

### Measures

#### Cannabis Use

At each assessment, participants reported past 6-month use and past 30-day use of 5 cannabis products using questions derived from validated national survey items.^3^ Cannabis products included “(1) combustible cannabis (eg, pot, weed, hash, reefer, or bud); (2) blunts (ie, cannabis rolled in tobacco leaf or cigar casing); (3) electronic device to vape cannabis or hash oil (eg, liquid pot, weed pen) [vaporized cannabis]; (4) cannabis or THC food or drinks (eg, pot brownies, edibles, butter, oil) [edible cannabis]; and (5) dabbing (eg, wax, shatter, budder, butane hash oil, BHO) [cannabis concentrate].” Past 6-month use was assessed by a dichotomous response set (0 indicated no and 1 indicated yes), and frequency of use in the past 30 days was assessed by an ordinal item with 9 options ranging from 1, indicating 0 days, to 9, indicating all 30 days. These response categories were recoded into quantitative count variables by taking the mean value of each ordinal category (ie, rounding up to the nearest integer for negative binomial regression models) as follows: 0 days (0 days), 2 days (1-2 days), 4 days (3-5 days), 8 days (6-9 days), 12 days (10-14 days), 17 days (15-19 days), 22 days (20-24 days), 27 days (25-29 days), and 30 days (all 30 days).

#### Covariates

To address possible confounding influences, baseline factors previously associated with cannabis use were included as covariates.^16^ Self-reported sociodemographic covariates (ie, age, gender, race/ethnicity, parental education level, and living situation), family history of drug use (ie, cigarette use, alcohol use disorder, and drug use disorder), and other substance use by participants (ie, past 30-day use of alcohol, combustible cigarettes, e-cigarettes with and without nicotine, hookahs, big or little cigars, stimulants, prescription stimulants, and prescription painkillers) were assessed with yes/no questions at baseline. The following measures were also assessed using validated self-report scales and are detailed in the eAppendix 1 in the Supplement: delinquent behavior,^17^ depressive symptoms,^18,19^ and attention-deficit/hyperactivity disorder.^20,21^

### Statistical Analysis

We assessed the association of baseline past 30-day cannabis product use (use of a given product on 0 vs 1-2 days of the past 30 days) with (1) past 6-month use of that product (yes or no; binary logistic regression models) and (2) number of days that product was used in the past 30 days (number of days [0-30]; negative binomial regression models), averaged across the 6-month and 12-month follow-up surveys using multilevel models that included all cannabis products as simultaneous factors in a single model. Odds ratios (ORs) or rate ratios (RRs) and 95% CIs of a single estimate of the average association of the baseline regressor with outcome data at the 6-month and 12-month follow-up surveys are reported. Separate models were fit for each cannabis product outcome. Primary results focused on each prospective association of a given product at baseline with the same product at follow-up surveys (eg, combustible cannabis use at baseline and combustible cannabis use at follow-up). The χ^2^ difference test, using log-likelihood values with (vs without) equality constraints on the association between 5 cannabis products, was used to determine whether the strength of associations estimated in regression models differed among the products (eTable 1 in the Supplement). Sensitivity analyses are described in the eAppendix 1 in the Supplement. Analyses were tested in Mplus version 7 (Muthén & Muthén) using 2-level random effects, as follows: time was nested within students, and school-level random effects were included to account for clustering of students within schools. Missing data were managed with full information maximum likelihood estimation. Benjamini-Hochberg multiple-testing corrections were used to maintain an overall α of .05.^22^ Statistical significance was set at *P* < .05, and all tests were 2-tailed.

## Results

### Study Sample

The flowchart describing the final analytic sample is presented in the Figure. Among the 2685 students included in the analytic sample, 1477 (55.0%) were young women, the mean (SD) age was 17.1 (0.4) years, and a plurality of the sample (1231 [46.6%]) consisted of Hispanic adolescents, with the remainder of the sample including 498 (18.8%) Asian adolescents, 127 (4.8%) African American adolescents, 423 (16.0%) white adolescents, and 363 (13.7%) adolescents with another ethnicity (Table 1). Few youth reported parent(s) without a high school diploma (286 [12.3%]), and most youth lived with both parents (1781 [66.9%]). Family history of substance use was common (1735 [67.5%]). Nearly half of the sample reported depressive symptoms (1197 [45.0%]), while few (158 [6.7%]) reported symptoms of attention-deficit/hyperactivity disorder. In this sample of participants with no cannabis use or experimental cannabis use, 505 youths (18.8%) reported past 30-day use of any noncannabis substance at baseline. The analytic sample did not vary appreciably from those excluded from analysis (eAppendix 2 in the Supplement).

**Table 1.** Descriptive Characteristics of the 2685 Participants at Baseline

Characteristic	No. (%)^a^
Women	1477 (55.0)
Age, mean (SD), y	17.10 (0.40)
Race/ethnicity	
Hispanic	1231 (46.6)
Asian	498 (18.8)
African American	127 (4.8)
White	423 (16.0)
Other^b^	363 (13.7)
Parent(s) without high school diploma^c^	286 (12.3)
Living with both parents vs other^d^	1781 (66.9)
Family substance use history^e^	1735 (67.5)
Depressive symptoms^f^	1197 (44.8)
Attention-deficit/hyperactivity disorder^g^	158 (6.7)
Delinquent behaviors, mean (SD)^h^	13.96 (4.11)
Past 30-d use of noncannabis substances^i^	
None	2180 (81.2)
Any	505 (18.8)
Combustible cannabis use, d	
0	2524 (94.1)
1-2	158 (5.9)
Blunt use, d	
0	2591 (96.6)
1-2	90 (3.4)
Vaporized cannabis use, d	
0	2667 (99.4)
1-2	17 (0.6)
Edible cannabis use, d	
0	2606 (97.1)
1-2	78 (2.9)
Concentrate cannabis use, d	
0	2667 (99.4)
1-2	15 (0.6)
Age at initiation of any cannabis use, mean (SD), y^j^	15.46 (1.77)

^a^ Available (nonmissing) data ranges from 2327 to 2685 participants.

^b^ Other race/ethnicity includes multiracial, American Indian or Alaska Native, Native Hawaiian or Pacific Islander, and other races.

^c^ The 358 students who did not respond to the survey question or who marked “don’t know” were not included in the denominator.

^d^ Other category included living with mother or father only, with stepparent(s), in a group home, and with someone else.

^e^ Any vs no history of family members’ smoking cigarettes, alcohol use disorder, or drug use disorder.

^f^ Screened positive for mild to moderate depressive symptoms or higher on the Center for Epidemiologic Studies Depression Scale (mean [SD] score, 15.72 [12.32]).

^g^ Symptom positive to either category of the Attention-Deficit/Hyperactivity Disorder Self-rating Scale.

^h^ Score ranges from 11 to 66, with higher scores indicating greater frequency of engaging in 11 different delinquent behaviors (1 indicates never and 6 indicates ≥10 times).

^i^ Past 30-day use of noncannabis products includes alcohol, combustible cigarettes, e-cigarettes (with or without nicotine), hookahs, cigars (big or little), stimulants, prescription stimulants, and prescription painkillers.

^j^ Data available data for 214 students who used any cannabis products in the past 30 days at baseline.

### Cannabis Use

Among participants in the analytic sample, 158 (5.9%) reported combustible cannabis use on 1 to 2 of the past 30 days at baseline, 90 (3.4%) reported blunt use, and 78 (2.9%) reported edible cannabis use. Few participants reported vaping cannabis (17 [0.6%]) or using cannabis concentrates (15 [0.6%]) (Table 1).

### Past 6-Month Cannabis Use

Overall, the prevalence of past 6-month use of a given cannabis product was higher for those who had used that product on 1 to 2 (vs 0) days at baseline (Table 2). Larger differences in the absolute prevalence of past 6-month use at follow-up for those reporting 1 to 2 (vs 0) days were observed for combustible cannabis (absolute difference, 56.9%; 95% CI, 53.0%-60.8%) and blunts (absolute difference, 54.3%; 95% CI, 49.2%-59.4%) than for vaporized cannabis, edible cannabis, or cannabis concentrate use (vaporized cannabis use: absolute difference, 30.3%; 95% CI, 21.4%-39.1%; edible cannabis use: absolute difference, 33.9%; 95% CI, 29.0%-38.9%; cannabis concentrate use: absolute difference, 40.3%; 95% CI, 32.7%-47.9%).

**Table 2.** Association of Baseline Use of Each Cannabis Product With Past 6-Month Use and Number of Days of Use in Past 30 Days at 6-Month and 12-Month Follow-ups

Baseline Cannabis Use	Past 6-mo Use at 6-mo and 12-mo Follow-ups			Use in Past 30 d at 6-mo and 12-mo Follow-ups, d		
	Prevalence, %	Absolute Difference (95% CI)	OR (95% CI)^a^	Mean (SD)	Absolute Difference (95% CI)	RR (95% CI)^b^
Combustible cannabis, d						
0	16.1	0 [Reference]	1 [Reference]	0.56 (2.78)	0 [Reference]	1 [Reference]
1-2	73.1	56.9 (53.0-60.8)	6.01 (3.66-9.85)^c^^,^^d^	3.07 (5.97)	2.52 (2.17-2.87)	2.81 (1.78-4.42)^c^^,^^e^
Blunts, d						
0	12.3	0 [Reference]	1 [Reference]	0.33 (2.02)	0 [Reference]	1 [Reference]
1-2	66.7	54.3 (49.2-59.4)	2.77 (1.45-5.29)^d^	2.29 (5.17)	1.96 (1.63-2.30)	1.59 (0.76-3.31)
Vaporized cannabis, d						
0	6.4	0 [Reference]	1 [Reference]	0.21 (1.81)	0 [Reference]	1 [Reference]
1-2	36.7	30.3 (21.4-39.1)	5.34 (1.51-11.20)^c^^,^^f^	0.87 (2.15)	0.66 (0.05-1.26)	2.14 (0.45-10.30)^d^
Edible cannabis, d						
0	9.7	0 [Reference]	1 [Reference]	0.25 (1.92)	0 [Reference]	1 [Reference]
1-2	43.6	33.9 (29.0-38.9)	3.32 (1.86-5.95)^c^	1.38 (4.16)	1.13 (0.80-1.46)	1.68 (0.84-3.36)
Cannabis concentrate, d						
0	4.1	0 [Reference]	1 [Reference]	0.11 (1.34)	0 [Reference]	1 [Reference]
1-2	44.4	40.3 (32.7-47.9)	5.87 (1.18-23.80)^c^^,^^d^	2.59 (5.89)	2.48 (1.95-3.01)	9.42 (2.02-35.50)^c^^,^^e^

Abbreviations: OR, odds ratio; RR, rate ratio.

^a^ Binary logistic regression models for respective outcome adjusted for 5 past 30-day use of cannabis product regressors (ie, combustible, blunts, vaporized, edible, and concentrated), with the time variable, participants’ gender, age, race/ethnicity, parental education level, living situation, family substance use history, past 30-day noncannabis product use, delinquent behaviors, depressive symptoms, and attention-deficit/hyperactivity disorder at baseline as simultaneous regressors.

^b^ Negative binomial regression models for respective outcome adjusted for 5 past 30-day use of cannabis product regressors (ie, combustible, blunts, vaporized, edible, and concentrated), with the time variable, participants’ gender, age, race/ethnicity, parental education level, living situation, family substance use history, past 30-day noncannabis product use, delinquent behaviors, depressive symptoms, and attention-deficit/hyperactivity disorder at baseline as simultaneous regressors.

^c^ Statistically significant after Benjamini-Hochberg corrections for multiple testing to control false-discovery rate at .05 (based on 2-tailed corrected *P* value).

^d^ In post hoc pairwise contrast for χ^2^ difference tests, ORs for combustible cannabis and cannabis concentrate showed statistical different than those for blunts and edible cannabis. Tests were conducted using the log-likelihood values with the maximum likelihood robust estimator (χ^2^_1_ > 3.84).

^e^ In post hoc pairwise contrast for χ^2^ difference tests, the RR for cannabis concentrate showed statistical difference with all other groups. Tests were conducted using the log-likelihood values with the maximum likelihood robust estimator (χ^2^_1_ > 3.84).

^f^ In post hoc pairwise contrast for χ^2^ difference tests, the OR for vaporized cannabis showed no statistical difference with any other group. Tests were conducted using the log-likelihood values with the maximum likelihood robust estimator (χ^2^_1_ > 3.84).

In models adjusted for demographic characteristics and poly–cannabis product use, use of any product on 1 to 2 (vs 0) days at baseline was associated with significantly greater odds of reporting past 6-month use of that product at follow-ups (Table 2). Comparatively stronger associations of baseline use with subsequent past 6-month use at the 6-month and 12-month follow-ups were observed for combustible cannabis use (OR, 6.01; 95% CI, 3.66-9.85) and cannabis concentrate use (OR, 5.87; 95% CI, 1.18-23.80) than for blunts (OR, 2.77; 95% CI, 1.45-5.29) or edible cannabis use (OR, 3.32; 95% CI, 1.86-5.95) (*P* for comparison < .05). Results for vaporized cannabis use (OR, 5.34; 95% CI, 1.51-11.20) were not significantly different from results for the other products (Table 2).

### Frequency of Past 30-Day Cannabis Use

The number of days of use of a product at follow-up was higher for those who had used a given product on 1 to 2 vs 0 days at baseline (Table 2). Larger absolute differences in the number of days of use reported at follow-up by baseline use pattern (1-2 vs 0 days) were observed for combustible cannabis use (absolute difference, 2.52 days; 95% CI, 2.17-2.87 days), blunts (absolute difference, 1.96 days; 95% CI, 1.63-2.30 days), and cannabis concentrate (absolute difference, 2.48 days; 95% CI, 1.95-3.01 days) compared with edible cannabis use (absolute difference, 1.13 days; 95% CI, 0.80-1.46 days) and vaporized cannabis use (absolute difference, 0.66 days; 95% CI, 0.05-1.26 days).

However, in regression models adjusting for demographic characteristics and poly–cannabis product use, the association of cannabis use at baseline with the mean number of days of use across 6- and 12-month follow-up surveys was significantly stronger for cannabis concentrate (RR, 9.42; 95% CI, 2.02-35.50) than for the use of any other cannabis product (*P* for comparison < .05) (Table 2). While combustible cannabis use on 1 to 2 (vs 0) days at baseline was associated with use on approximately 2.8 more days in the past 30 days at follow-up (RR, 2.81; 95% CI, 1.78-4.42), no significant associations were observed for blunts, vaporized, or edible cannabis in models adjusting for demographic characteristics and poly–cannabis product use. Sensitivity analyses are described in eAppendix 2 and eTable 2 in the Supplement.

### Cross-Product Associations

Participants who reported use of combustible cannabis on 1 to 2 (vs 0) days in the past 30 days at baseline had increased odds of past 6-month use of all other cannabis products in models adjusted for poly–cannabis product use and demographic characteristics (blunt use: OR, 3.85; 95% CI, 2.39-6.21; *P* < .001; vaporized cannabis use: OR, 2.43; 95% CI, 1.35-4.38; *P* = .003; edible cannabis use: OR, 2.68; 95% CI, 1.59-4.53; *P* < .001; cannabis concentrate use: OR, 2.87; 95% CI, 1.23-6.69; *P* = .01) (eTable 3 in the Supplement). Use of any of the other cannabis products was only associated with subsequent use of the same product in adjusted models, with the exception of edible cannabis. Participants who used edible cannabis on 1-2 (vs 0) days also had higher odds of combustible cannabis use across follow-ups (OR, 2.32; 95% CI, 1.23-4.39; *P* = .01). In analyses investigating the association with number of days of use at follow-up, combustible cannabis use at baseline (1-2 vs 0 days) was associated with using each of the other products (except cannabis concentrate) on more days in the past month on average, across 6- and 12-month follow-ups, after adjusting for demographic characteristics and poly–cannabis product use (blunt use: RR, 2.76; 95% CI, 1.59-4.78; *P* < .001; vaporized cannabis use: RR, 2.58; 95% CI, 1.08-6.13; *P* = .02; edible cannabis use: RR, 2.37; 95% CI, 1.28-4.39; *P* = .006). No other cross-product associations were observed (eTable 4 in the Supplement).

## Discussion

This study provides new evidence that the probability of persistent use of cannabis and progression in the number of days of cannabis use over 1 year of follow-up differs among 5 cannabis products. Specifically, this study found stronger associations of persistent cannabis use for combustible cannabis and cannabis concentrate than for blunt or edible cannabis in models adjusted for poly–cannabis product use and demographic characteristics. Stronger associations of progression of cannabis use were observed for cannabis concentrate than for any other cannabis product in models adjusted for poly–cannabis product use and demographic characteristics. Together, these findings suggest that concentrated and combustible cannabis use may carry a higher risk of continued and more frequent cannabis use among adolescents who are in the early stages of experimentation and have not yet progressed to more frequent or heavy cannabis use patterns. A unique strength of this study was the evaluation of associations of light use with persistence and progression of use among 5 cannabis products and statistical control of concomitant use of multiple products, permitting inferences regarding the comparative risk associated with use of a single product vs another.

The differential risk of persistent for use of combustible cannabis and cannabis concentrate (vs blunt or edible cannabis) and of progression for cannabis concentrate (vs all other products) could be explained by several mechanisms. First, cannabis products that are easier to access may be more likely to be used—and continue to be used—by adolescents who are unable to legally purchase recreational cannabis products in the United States.^4,11,12^ Thus, the stronger association of combustible cannabis use with continued past 6-month use in follow-ups could be the result of ease of access to combustible cannabis. Cannabis in combustible form is the most commonly used cannabis product^4^ and is thus also likely the easiest for adolescents to access.^13^ However, those using combustible cannabis at baseline also had increased odds of reporting past 6-month use of any other cannabis product, suggesting that it may instead serve as an easier entry into cannabis use.

Ease of access is unlikely to explain the associations of cannabis concentrate use, given the increased price point and the complex equipment needed to use it.^8,23^ An alternative explanation for these findings could be that cannabis concentrate may deliver a stronger and faster dose of THC than other cannabis products.^7,8^ Levels of THC in cannabis concentrate are 2 to 4 times stronger than those in combustible cannabis and can reach concentrations greater than 80%, delivering a very high effective THC dose to the user.^2^ High levels of THC delivered to the user may increase the positive sensory and pharmacological experience associated with early use of cannabis concentrate and early development of dependent symptoms associated with this experience. Youth may seek out and use cannabis concentrate products despite challenges with access to cannabis in this form either because they are interested in continued experiences with the sensory and pharmacological effects of cannabis concentrate specifically or because they have developed dependence owing to high levels of exposure to THC via use of cannabis concentrate.

The comparatively lower, albeit still elevated, risk of persistence and progression of cannabis use among light experimenters for blunts, vaporized cannabis, and edible cannabis may be because of several relevant and associated factors. These products may be more difficult to obtain, may lead to a less desirable high (including adverse effects of use, such as nausea, vomiting, or hallucinations/delusions, which are commonly reported among those who have experimented with edible cannabis products^9^), or may have a lower use disorder potential because lower levels of THC are delivered and/or THC is delivered more slowly, resulting in a less immediate physiological effect and thereby lessening the reinforcing effect that occurs with more potent cannabis products.

The potential differential use disorder risk conferred by different cannabis products may warrant a public health response targeting specific cannabis products. While cannabis concentrate was associated with greater number of days of use and greater likelihood of past 6-month use across follow-ups, the overall prevalence of cannabis concentrate and number of days of use at baseline and follow-up were relatively low in comparison with other cannabis products. In contrast, combustible cannabis conferred a high risk of persistent use and was used at a higher rate compared with other products at baseline and across both follow-ups. As such, prevention efforts targeting combustible cannabis use among adolescents may result in greater overall public health consequences compared with efforts that target cannabis concentrate. However, the legalization and commercialization of cannabis products are likely to expand the overall cannabis market and accessibility of a more diverse array of cannabis products, with differing use disorder potential, so continued monitoring of the ongoing trends in the use of different products among susceptible populations is needed. At present, targeted prevention campaigns are nevertheless needed to reduce youth cannabis use overall and to reduce the risk of transition to heavier patterns of use that confer greater health risks.

### Limitations

This study was subject to some limitations. The study included participants residing in southern California, where medicinal cannabis has been legal since 1996 and where adult-use (ie, recreational) cannabis was voted into law in 2016, with implementation and licensing of retailers effective in January 2018.^15^ As such, these findings may not be generalizable to other geographic locations within or outside the United States that have different regulatory environments. In addition, this study examined the change in cannabis use patterns from 11th to 12th grade, so findings may not be applicable to younger adolescents or to young adults. The number of days of use in the past 30 days at follow-up was relatively low for cannabis concentrate, particularly among those who had not used combustible cannabis at baseline, which was associated with use of all cannabis products at follow-up. There are inherent challenges in assessing dose of exposure to the psychoactive ingredient in cannabis products, THC, particularly among various forms of administration and various products. As a result, we were unable to account for differences in THC dose by cannabis product, which may have influenced the risk of persistence or progression. Future studies should aim to develop and validate measures of dose and duration of use. It is possible that the differences between products were confounded by unmeasured factors or selection bias whereby youth drawn to a particular product (eg, cannabis concentrate) vs another (eg, edible cannabis) were at a preexisting high risk of becoming persistent or frequent users. We addressed this potential analytically by including a number of potential confounders that may be associated with cannabis use and with use of a particular product compared with another. In addition, the use of a single model with all baseline cannabis product use variables, which allowed for relative comparisons between different products, minimized the potential that significant results for a particular product vs another product could be explained simply by a high risk of any cannabis use among poly–cannabis product users.

Furthermore, we aimed to examine the risk of persistence and progression of cannabis use by type of product used among experimenters; frequent users reporting use on at least 3 to 5 days of the past 30 days at baseline were excluded from analyses. This group likely represents a mix of participants who are early in their use and on an upward trajectory, youth who are consistently light users, and youth who may have recently decreased their cannabis use (and thus were previously a frequent user). We conducted sensitivity analyses to better define this sample. When we restricted the sample to those with no history of frequent use on the survey before the baseline survey in this study, results were generally similar. Notably, we did not have data on cannabis concentrate use in the past 30 days for the prior survey and thus could not restrict prior frequent cannabis concentrate users. Although cannabis use in adolescence is generally unstable with nonnegligible natural variation in use patterns, our findings nevertheless suggest that the type of product used among current light users is prognostic of persistent cannabis use and progression to more frequent use.

## Conclusions

Overall, the persistence of cannabis use was stronger for combustible cannabis and cannabis concentrate than for blunts or edible cannabis, and the association of progression to higher levels cannabis use was stronger for cannabis concentrate than for any other product, suggesting that combustible cannabis and cannabis concentrate may carry a higher potential for use disorder. Our findings suggest that combustible cannabis and cannabis concentrate should be targeted in prevention campaigns to reduce the rates of progression to heavy cannabis use in adolescent populations and the adverse health effects that have been associated with heavy cannabis use early in life.

## Notes

Supplement. **eAppendix 1.** Additional Description of Methods **eAppendix 2.** Supplementary Results **eTable 1.** Descriptive Statistics of Study Covariates of Students Included in (vs Excluded From) the Primary Analytic Sample **eTable 2.** Association of Baseline Use of Each Cannabis Product With Past 6-Month Use and Number of Days That Product Was Used in the Past 30 Days at 6-Month and 12-Month Follow-ups **eTable 3.** Adjusted Models Indicating Cross-Product and Covariate Associations With Past 6-Month Use Outcomes **eTable 4.** Adjusted Models Indicating Cross-Product and Covariate Associations With Past 30-Day Use Outcomes
