# Supplementary material for: Risk of Persistence and Progression of Use of 5 Cannabis Products After Experimentation Among Adolescents
Source: JAMA Netw Open. 2020 Jan 24;3(1):e1919792. doi: 10.1001/jamanetworkopen.2019.19792 (PMC6991277; doi:10.1001/jamanetworkopen.2019.19792)
Supplement: Supplement. — eAppendix 1. Additional Description of Methods eAppendix 2. Supplementary Results eTable 1. Descriptive Statistics of Study Covariates of Students Included in (vs Excluded From) the Primary Analytic Sample eTable 2. Association of Baseline Use of Each Cannabis Product With Past 6-Month Use and Number of Days That Product Was Used in the Past 30 Days at 6-Month and 12-Month Follow-ups eTable 3. Adjusted Models Indicating Cross-Product and Covariate Associations With Past 6-Month Use Outcomes eTable 4. Adjusted Models Indicating Cross-Product and Covariate Associations With Past 30-Day Use Outcomes [file jamanetwopen-3-e1919792-s001.pdf]

Supplementary Online Content

Barrington-Trimis JL, Cho J, Ewusi-Boisvert E, et al. Risk of persistence and progression of use of 5 cannabis products after experimentation among adolescents. *JAMA Netw Open*. 2020;3(1):e1919792. doi:10.1001/jamanetworkopen.2019.19792

eAppendix 1. Additional Description of Methods

eAppendix 2. Supplementary Results

eTable 1. Descriptive Statistics of Study Covariates of Students Included in (vs Excluded From) the Primary Analytic Sample

eTable 2. Association of Baseline Use of Each Cannabis Product With Past 6-Month Use and Number of Days That Product Was Used in the Past 30 Days at 6-Month and 12-Month Follow-ups

eTable 3. Adjusted Models Indicating Cross-Product and Covariate Associations With Past 6-Month Use Outcomes

eTable 4. Adjusted Models Indicating Cross-Product and Covariate Associations With Past 30-Day Use Outcomes

This supplementary material has been provided by the authors to give readers additional information about their work.

© 2020 Barrington-Trimis JL et al. *JAMA Network Open.*

eAppendix 1. Additional Description of Methods

Covariates

Delinquent behavior was assessed with an 11-item measure of past 6-month delinquent behaviors (e.g., stealing, destroying property, physically fighting, being suspended from school).[20] The frequencies of 11 behavior items ranging from 1 (“Never”) to 6 (“10 or more times”) were averaged (α=0.79). Depressive symptomology was assessed with the Center for Epidemiologic Studies Depression Scale (CESD).[21] The CESD is a 20-item measure of depressive symptoms experienced over the past week, rated on a 4-point Likert scale, ranging from 0 (Rarely or None of the time; 0–1 days) to 3 (Most or all of the time; 5–7 days). The total sum score of the 20 items was computed (Cronbach’s α = 0.81) and dichotomized (yes/no) based on the measure’s recommended cut-off for clinical depression.[22] Attention deficit/hyperactivity disorder (ADHD) was assessed using the DSM-IV 18-item Current Symptoms Scale-Self Report Form measure[23] (α = 0.90), including 9 inattention symptoms (e.g., difficulty organizing and completing tasks) and 9 hyperactivity-impulsivity symptoms (e.g., trouble remaining still or with task persistence). Respondents rated the frequency that they experienced each symptom in the preceding 6 months, ranging from 0 (never or rare) to 3 (very often). Consistent with recommendations and DSM-IV criteria,[23,24] adolescents who reported experiencing 6 more symptoms in either inattention or hyperactivity-impulsivity categories were classified as having ADHD symptoms over the past 6 months.

Sensitivity analysis

We conducted additional sensitivity analyses to investigate the robustness of results under different assumptions regarding the characterization of experimental users. 1) We

© 2020 Barrington-Trimis JL et al. *JAMA Network Open.*

additionally restricted the sample to participants who had not used any cannabis product on 3-5 or more of the past 30 days at the wave prior to the baseline wave in the current study (N=80; 3.0% of the sample) to estimate associations among experimental users who were not recent heavy cannabis users. The number of users who were excluded ranged from N=3 (17.6% of vaporized users) to N=14 (9.7% of combustible users). 2) We additionally created a three level exposure variable that included never use, lifetime use but no use in the past 30 days, use on 1-2 of the past 30 days in order to directly compare use on 1-2 of the past 30 days to no prior use of that cannabis product.

© 2020 Barrington-Trimis JL et al. *JAMA Network Open.*

eAppendix 2. Supplementary Results

Study Sample

Of the 3396 participants who were enrolled in the study in the Fall of 9th grade, 3065 participants provided data for at least one cannabis product at the Fall of 11th grade data collection (baseline; See Figure 1). 355 participants were excluded from the study due to heavy use of any cannabis product (use on 3-5 or more of the past 30 days) at baseline. An additional 25 participants were excluded due to missing information on past 30-day cannabis use outcomes at both 6 and 12 month follow-ups, resulting in a final analytic sample of 2685 participants.

The analytic sample varied from the sample of participants excluded for no information on cannabis use at baseline (N=331), from participants excluded for heavy cannabis use at baseline (N=355), and from participants excluded because they had not completed questions assessing cannabis use at both the 6- and 12-month follow-ups (see eTable 1). In general, the analytic sample had more females, participants were slightly younger, fewer participants were Hispanic and more participants were Asian, fewer participants had parent(s) without a high school diploma, more participants lived with both parents, and prevalence of a history of family substance use was lower.

Sensitivity Analyses

Associations in the restricted sample generally differed by no more than 20% from those reported in Table 2 with two exceptions where the strength of the association increased - 1) the association of vaporized use with prevalence of past 6 month use at follow-up increased by about 44% (OR=7.70; 95%CI: 1.77, 23.4); 2) the association of edible use with number of days of use at follow-up increased by about 38% (RR=2.32; 95%CI:

© 2020 Barrington-Trimis JL et al. *JAMA Network Open.*

1.22, 4.43; data not tabulated). In evaluation of associations of 1-2 days of use (vs. never use) with prevalence and progression, the pattern of associations generally did not change, though the strength of each association was generally stronger (see eTable 2).

eTable 1. Descriptive Statistics of Study Covariates of Students Included in (vs Excluded From) the Primary Analytic Sample

	**Students enrolled in cohort in 9th grade (N=3396)**				
**Baseline Variables**	**Analytic sample (N=2685)[a]**	**Excluded for no information of all past 30-day cannabis use items at baseline (N=331)[b]**	**Excluded for heavy use of any cannabis product in the past 30 days at baseline (N=355)[c]**	**Excluded for no information of past 30-day cannabis product outcomes across follow-ups (N=25)[d]**	**Test of group differences[e]**
	**N (%) / Mean (*SD*)**	**N (%) / Mean (*SD*)**	**N (%) / Mean (*SD*)**	**N (%) / Mean (*SD*)**	**P**
Female (vs. Male), N (%)	1477 (55.0)[k]	142 (43.0)[l]	182 (51.4)[k,l]	12 (48.0)[k,l]	<.001
Age, Mean (*SD*), y	17.10 (0.40)[l]	17.36 (0.45)[k]	17.19 (0.45)[l]	17.23 (0.47)[k,l]	<.001
Race/ethnicity, N (%)					<.001
Hispanic	1231 (46.6)[l]	177 (55.8)[k]	185 (53.8)[k,l]	12 (52.2)[k,l]	
Asian	498 (18.8)[k]	28 (8.8)[l]	31 (9.0)[l]	3 (13.0)[k,l]	
African American	127 (4.8)[k]	26 (8.2)[k]	13 (3.8)[k]	0 (0.0)[k]	
White	423 (16.0)[k,l]	43 (13.6)[l]	74 (21.5)[k]	4 (17.4)[k,l]	
Other[f]	363 (13.7)[k]	43 (13.6)[k]	41 (11.9)[k]	4 (17.4)[k]	
Parent(s) without high school diploma, N (%)[g]	286 (12.3)[k]	50 (18.7)[k]	46 (14.6)[k,l]	1 (4.5)[k,l]	.01
Living with both parents (vs. Other), N (%)[h]	1781 (66.9)[k]	144 (44.6)[m]	194 (55.6)[l]	15 (60.0)[k,l,m]	<.001
Family substance use history, N (%)[i]	1735 (67.5)[l]	221 (75.7)[k,l]	275 (81.4)[k]	17 (73.9)[k,l]	<.001

Note. [a]Available data (Ns = 2327 – 2685). [b]Available data (Ns = 268 – 331). [c]Available data (Ns = 314 – 355). [d]Available data (Ns = 22 – 25).
[e]Calculated using the χ^2 test for categorical variables and using one-way Analysis of Variance (ANOVA) for continuous variables. Groups not sharing superscript numerals are significantly different in Bonferroni-corrected post-hoc pairwise contrasts for χ^2 tests and ANOVA Least Significant Difference.
[f]Other race/ethnicity includes multiracial, American Indian or Alaska Native, Native Hawaiian or Pacific Islander, and other races.
[g]Students who did not respond to the survey question or who marked "don't know" are not included in the denominator.
[h]Other category includes living with mother/father only, living with step parent(s), group home, and someone else.
[i]Any (vs. No) history of family members' smoking cigarettes, alcohol abuse problems, and drug abuse problems.
[j]Past 30-day use of non-cannabis products including alcohol, combustible cigarettes, e-cigarette (with/without nicotine), hookah, cigars (big/little), stimulants, prescription stimulants, prescription painkillers.
[k,l,m]Groups not sharing superscript numerals in each row are significantly different in post-hoc pairwise contrast for Chi-square difference tests, which were conducted using the loglikelihood values with the maximum likelihood robust estimator (χ^2/1df > 3.84).

© 2020 Barrington-Trimis JL et al. *JAMA Network Open.*

eTable 2. Association of Baseline Use of Each Cannabis Product With Past 6-Month Use and Number of Days That Product Was Used in the Past 30 Days at 6-Month and 12-Month Follow-ups

**Baseline Regressors**	**Prevalence**	**Past 6 month use at 6- and 12-month follow-up[a]**	**Number of days used in the past 30 days at 6-and 12-month follow-up[b]**
	**N (%)**	**OR (95%CI)**	**RR (95%CI)**
**Combustible**			
Never user	1871 (69.8)	Ref	Ref
Lifetime use, but no use in past 30 days	653 (24.3)	3.32 (2.29, 4.84)	2.09 (1.31, 5.34)
Use on 1-2 of past 30 days	158 (5.9)	9.70 (5.48, 17.24)	4.49 (2.55, 7.88)
**Blunts**			
Never user	2077 (77.5)	Ref	Ref
Lifetime use, but no use in past 30 days	514 (19.2)	1.05 (0.71, 1.58)	1.06 (0.35, 3.37)
Use on 1-2 of past 30 days	90 (3.4)	2.93 (1.48, 5.83)	2.67 (1.29, 5.56)
**Vaporized**			
Never user	2377 (88.6)	Ref	Ref
Lifetime use, but no use in past 30 days	290 (10.8)	1.19 (0.75, 1.89)	1.13 (0.44, 7.16)
Use on 1-2 of past 30 days	17 (0.6)	6.48 (1.66, 23.22)	2.75 (0.51, 14.68)
**Edible**			
Never user	2092 (77.9)	Ref	Ref
Lifetime use, but no use in past 30 days	514 (19.2)	1.13 (0.73, 1.75)	0.98 (0.45, 1.98)
Use on 1-2 of past 30 days	78 (2.9)	3.69 (1.41, 7.14)	1.82 (0.56, 4.91)
**Concentrate**			
Never user	2591 (96.6)	Ref	Ref
Lifetime use, but no use in past 30 days	76 (2.8)	1.15 (0.54, 2.44)	1.72 (0.56, 5.34)
Use on 1-2 of past 30 days	15 (0.6)	9.37 (1.55, 26.62)	12.93 (1.75, 47.72)

Note. N=2685.

[a]Binary logistic regression models for respective outcome adjusting for five past 30-day cannabis product use regressors (i.e., combustible, blunts, vaporized, edible, concentrate), the time variable, participants' sex, age, race/ethnicity, parental education level, living situation, family substance use history, past 30-day non-cannabis product use, delinquent behaviors, depressive symptom, and ADHD at baseline, as simultaneous regressors.

[b]Negative binomial regression models for respective outcome adjusting for five past 30-day cannabis product use regressors (i.e., combustible, blunts, vaporized, edible, concentrate), the time variable, participants' sex, age, race/ethnicity, parental education level, living situation, family substance use history, past 30-day non-cannabis product use, delinquent behaviors, depressive symptom, and ADHD at baseline, as simultaneous regressors.

© 2020 Barrington-Trimis JL et al. *JAMA Network Open.*

eTable 3. Adjusted Models Indicating Cross-Product and Covariate Associations With Past 6-Month Use Outcomes

	**Outcomes: Past 6-month use prevalence outcomes across follow-ups[a]**									
**Baseline regressors**	**Combustible**		**Blunts**		**Vaporized**		**Edible**		**Concentrate**	
	**OR (95%CI)**	**P**	**OR (95%CI)**	**P**	**OR (95%CI)**	**P**	**OR (95%CI)**	**P**	**OR (95%CI)**	**P**
Female (vs. Male)	0.88 (0.70, 1.10)	.24	0.83 (0.65, 1.06)	.14	0.79 (0.57, 1.09)	.16	0.76 (0.59, 0.99)	.04	0.65 (0.43, 0.97)	.03
Age[b]	1.08 (0.96, 1.21)	.23	1.06 (0.93, 1.21)	.37	1.08 (0.92, 1.28)	.35	1.09 (0.96, 1.25)	.20	1.04 (0.84, 1.28)	.75
Race/ethnicity										
Asian	0.66 (0.45, 0.95)	.002	0.83 (0.55, 1.26)	.38	0.75 (0.43, 1.30)	.31	0.64 (0.40, 1.03)	.06	0.47 (0.20, 1.06)	.07
African American	0.99 (0.59, 1.66)	.98	1.32 (0.78, 2.24)	.30	0.57 (0.22, 1.45)	.24	1.01 (0.59, 1.72)	.97	0.69 (0.24, 1.99)	.49
White	0.99 (0.71, 1.39)	.96	1.03 (0.71, 1.51)	.87	1.26 (0.82, 1.94)	.30	0.90 (0.61, 1.33)	.59	1.50 (0.89, 2.55)	.13
Other[c]	0.97 (0.68, 1.35)	.80	1.05 (0.72, 1.54)	.79	0.86 (0.52, 1.43)	.55	0.90 (0.60, 1.34)	.61	0.82 (0.42, 1.59)	.55
Parent(s) without high school diploma[d]	0.76 (0.52, 1.12)	.17	0.82 (0.54, 1.24)	.34	0.96 (0.57, 1.63)	.88	0.84 (0.52, 1.23)	.32	0.82 (0.39, 1.65)	.55
Living with both parents[e]	0.79 (0.60, 0.96)	.02	0.75 (0.58, 0.97)	.03	0.83 (0.59, 1.63)	.27	0.80 (0.61, 1.04)	.10	1.12 (0.73, 1.69)	.61
Family substance use history[f]	1.23 (0.97, 1.58)	.09	1.19 (0.90, 1.56)	.22	1.41 (0.97, 2.06)	.07	1.24 (0.92, 1.67)	.16	1.00 (0.64, 1.55)	.99
Non-cannabis substance use[g]	2.81 (2.18, 3.62)	<.001	2.74 (2.08, 3.62)	<.001	2.17 (1.52, 3.10)	<.001	1.95 (1.44, 2.63)	<.001	2.05 (1.27, 3.31)	.003
Delinquent behaviors[b,h]	1.28 (1.12, 1.45)	<.001	1.25 (1.10, 1.42)	.001	1.22 (1.03, 1.45)	.02	1.27 (1.11, 1.44)	<.001	1.29 (1.07, 1.56)	.008
Depression[i]	1.14 (0.90, 1.44)	.27	1.02 (0.79, 1.32)	.86	0.96 (0.68, 1.34)	.72	1.10 (0.84, 1.45)	.48	1.02 (0.66, 1.57)	.95
ADHD[j]	1.44 (0.96, 2.16)	.08	1.14 (0.71, 1.83)	.59	1.38 (0.76, 2.53)	.30	1.31 (0.80, 2.13)	.28	1.71 (0.87, 3.37)	.12
Combustible (1-2 days vs. 0-days)[k]	6.01 (3.66, 9.85)	<.001	3.85 (2.39, 6.21)	<.001	2.43 (1.35, 4.38)	.003	2.68 (1.59, 4.53)	<.001	2.87 (1.23, 6.69)	.01
Blunts (1-2 days vs. 0-days)[k]	1.64 (0.81, 3.33)	.17	2.77 (1.45, 5.29)	.002	1.86 (0.89, 3.86)	.09	1.26 (0.63, 2.52)	.52	1.55 (0.78, 3.11)	.21
Vaporized (1-2 days vs. 0-days)[k]	2.58 (0.85, 7.81)	.09	2.03 (0.47, 8.74)	.34	5.34 (1.51, 11.17)	.009	2.14 (0.44, 10.34)	.34	3.85 (0.77, 12.34)	.10
Edible (1-2 days vs. 0-days)[k]	2.32 (1.23, 4.39)	.01	1.29 (0.65, 2.56)	.47	1.73 (0.89, 3.37)	.10	3.32 (1.86, 5.95)	<.001	0.63 (0.21, 1.87)	.41
Concentrate (1-2 days vs. 0-days)[k]	0.78 (0.10, 5.98)	.81	0.83 (0.14, 5.02)	.84	1.04 (0.16, 6.53)	.97	1.74 (0.50, 6.03)	.38	5.87 (1.18, 23.83)	.01
Time[l]	1.82 (1.57, 2.11)	<.001	1.83 (1.54, 2.18)	<.001	3.01 (2.29, 3.97)	<.001	1.88 (1.52, 2.32)	<.001	2.00 (1.43, 2.78)	<.001

Note. N=2685.
[a]Binary logistic regression models for respective past 6-month cannabis use outcome (yes/no) adjusting for all regressors simultaneously.
[b]Continuous scale regressors were standardized (Mean=0, *SD*=1).
[c]Other race/ethnicity includes multiracial, American Indian or Alaska Native, Native Hawaiian or Pacific Islander, and other races.
[d]Parent(s) without high school diploma vs. Parent(s) with high school diploma or higher education levels.
[e]Living with both parents vs. Other category (i.e., living with mother/father only, living with step parents, group home, and someone else). ‘
[f]Any (vs. No) history of family members' smoking cigarettes, alcohol abuse problems, and drug abuse problems.
[g]Past 30-day use of non-cannabis products including alcohol, combustible cigarettes, e-cigarette (with/without nicotine), hookah, cigars (big/little), stimulants, prescription stimulants, prescription painkillers.
[h]Continuous scale with higher scores indicating greater frequency of engaging in 11 different delinquent behaviors.
[i]Screen positive (vs. negative) for mild-to-moderate depressive symptoms or higher on the Center for Epidemiologic Studies Depression Scale.
[j]Symptom-positive to either category of the Attention/Deficit Hyperactivity Disorder Self-Rating Scale.
[k]Past 30-day cannabis product use (1-2 days vs. 0 days).
[l]Time continuous variable (scored at 6-month, 0 and 12-month, 1).
[j]Interaction terms between baseline cannabis regressors and time variable were tested in subsequent models, and no significant interaction effects were not detected across six models (P-values > .06).

© 2020 Barrington-Trimis JL et al. *JAMA Network Open.*

eTable 4. Adjusted Models Indicating Cross-Product and Covariate Associations With Past 30-Day Use Outcomes

	Outcomes: Past 30-day use frequency outcomes across follow-ups[a]									
**Baseline regressors**	**Combustible**		**Blunts**		**Vaporized**		**Edible**		**Concentrate**	
	**RR (95%CI)**	**P**	**RR (95%CI)**	**P**	**RR (95%CI)**	**P**	**RR (95%CI)**	**P**	**RR (95%CI)**	**P**
Female (vs. Male)	0.61 (0.43, 0.85)	.004	0.56 (0.37, 0.83)	.004	0.47 (0.26, 0.83)	.01	0.39 (0.25, 0.62)	<.001	0.35 (0.16, 0.78)	.01
Age[b]	1.03 (0.81, 1.30)	.81	1.01 (0.75, 1.37)	.93	0.97 (0.66, 1.42)	.86	1.01 (0.72, 1.40)	.99	1.05 (0.83, 1.31)	.73
Race/ethnicity										
Asian	0.71 (0.40, 1.25)	.24	0.83 (0.43, 1.59)	.56	0.43 (0.17, 1.09)	.07	0.37 (0.16, 0.86)	.02	0.26 (0.05, 1.54)	.14
African American	0.95 (0.38, 2.36)	.19	1.35 (0.47, 3.91)	.57	1.28 (0.15, 10.72)	.82	1.72 (0.47, 6.24)	.41	1.61 (0.20, 11.09)	.65
White	1.28 (0.76, 2.16)	.36	1.13 (0.53, 2.42)	.76	1.98 (0.81, 4.85)	.14	0.71 (0.32, 1.56)	.39	2.06 (0.69, 6.15)	.20
Other[c]	0.64 (0.33, 1.24)	.19	1.05 (0.44, 2.48)	.91	0.87 (0.26, 2.95)	.82	1.72 (0.47, 6.24)	.41	0.78 (0.32, 1.19)	.22
Parent(s) without high school diploma[d]	1.04 (0.56, 1.96)	.89	1.37 (0.66, 2.83)	.40	1.34 (0.43, 4.18)	.62	1.16 (0.48, 2.78)	.75	1.22 (0.24, 6.35)	.81
Living with both parents[e]	0.76 (0.50, 1.16)	.20	0.65 (0.40, 1.05)	.07	0.64 (0.33, 1.24)	.18	0.94 (0.53, 1.66)	.83	0.50 (0.20, 1.23)	.13
Family substance use history[f]	1.15 (0.75, 1.76)	.52	0.94 (0.54, 1.58)	.76	1.10 (0.49, 2.44)	.82	1.01 (0.53, 1.89)	.99	0.42 (0.18	
Non-cannabis substance use[g]	2.45 (1.69, 3.55)	<.001	2.15 (1.33, 3.48)	.002	1.36 (0.68, 2.73)	.38	1.41 (0.85, 2.36)	.19	2.17 (0.72, 6.59)	.17
Delinquent behaviors[b,h]	1.09 (0.89, 1.35)	.40	1.09 (0.84, 1.42)	.51	1.15 (0.81, 1.65)	.43	1.28 (0.99, 1.63)	.05	1.48 (1.06, 2.07)	.02
Depression[i]	0.98 (0.65, 1.44)	.88	0.90 (0.54, 1.43)	.51	0.72 (0.35, 1.19)	.20	0.95 (0.46, 1.96)	.89	0.92 (0.51, 1.40)	.48
ADHD[j]	2.14 (1.22, 3.75)	.008	1.55 (0.82, 2.93)	.18	1.04 (0.75, 1.53)	.74	1.05 (0.49, 2.06)	.87	2.45 (0.83, 7.32)	.11
Combustible (1-2 days vs. 0-days)[k]	2.81 (1.78, 4.42)	<.001	2.76 (1.59, 4.78)	<.001	2.58 (1.08, 6.13)	.02	2.37 (1.28, 4.39)	.006	1.46 (0.53, 5.62)	.43
Blunts (1-2 days vs. 0-days)[k]	1.33 (0.78, 2.25)	.29	1.59 (0.76, 3.31)	.22	2.14 (0.83, 5.50)	.11	1.92 (0.69, 5.34)	.21	2.39 (0.98, 8.08)	.05
Vaporized (1-2 days vs. 0-days)[k]	1.73 (0.42, 7.16)	.45	1.83 (0.32, 10.53)	.50	2.14 (0.45, 10.25)	.34	2.08 (0.24, 14.04)	.51	1.13 (0.16, 8.89)	.83
Edible (1-2 days vs. 0-days)[k]	1.24 (0.74, 2.09)	.41	0.58 (0.22, 1.30)	.16	1.31 (0.54, 3.14)	.55	1.68 (0.84, 3.36)	.14	0.99 (0.20, 4.85)	.99
Concentrate (1-2 days vs. 0-days)[k]	1.39 (0.43, 4.38)	.58	3.16 (0.43, 13.03)	.24	1.08 (0.14, 5.35)	.89	3.07 (0.31, 20.68)	.34	9.42 (2.02, 35.45)	.01
Time[l]	2.02 (1.51, 2.69)	<.001	1.98 (1.32, 2.99)	.001	2.54 (1.20, 5.35)	.01	1.94 (1.14, 3.30)	.01	1.37 (0.59, 3.18)	.47

Note. N=2685.
[a]Negative binomial regression models for respective past 30-day cannabis use outcome (range = 0 – 30) adjusting for all regressors simultaneously.
[b]Continuous scale regressors were standardized (Mean=0, *SD*=1).
[c]Other race/ethnicity includes multiracial, American Indian or Alaska Native, Native Hawaiian or Pacific Islander, and other races.
[d]Parent(s) without high school diploma vs. Parent(s) with high school diploma or higher education levels.
[e]Living with both parents vs. Other category (i.e., living with mother/father only, living with step parents, group home, and someone else).
[f]Any (vs. No) history of family members' smoking cigarettes, alcohol abuse problems, and drug abuse problems.
[g]Past 30-day use of non-cannabis products including alcohol, combustible cigarettes, e-cigarette (with/without nicotine), hookah, cigars (big/little), stimulants, prescription stimulants, prescription painkillers.
[h]Continuous scale with higher scores indicating greater frequency of engaging in 11 different delinquent behaviors.
[i]Screen positive (vs. negative) for mild-to-moderate depressive symptoms or higher on the Center for Epidemiologic Studies Depression Scale.
[j]Symptom-positive to either category of the Attention/Deficit Hyperactivity Disorder Self-Rating Scale.
[k]Past 30-day cannabis product use (1-2 days vs. 0 days).
[l]Time continuous variable (scored at 6-month, 0 and 12-month, 1).
[j]Interaction terms between baseline cannabis regressors and time variable were tested in subsequent models, and no significant interaction effects were not detected across six models (P-values > .25).

© 2020 Barrington-Trimis JL et al. *JAMA Network Open.*
